# Mitotic Spindle Proteomics in Chinese Hamster Ovary Cells

**DOI:** 10.1371/journal.pone.0020489

**Published:** 2011-05-27

**Authors:** Mary Kate Bonner, Daniel S. Poole, Tao Xu, Ali Sarkeshik, John R. Yates, Ahna R. Skop

**Affiliations:** 1 Department of Genetics, University of Wisconsin-Madison, Madison, Wisconsin, United States of America; 2 Department of Chemical Physiology, The Scripps Research Institute, La Jolla, California, United States of America; University of Texas-Houston Medical School, United States of America

## Abstract

Mitosis is a fundamental process in the development of all organisms. The mitotic spindle guides the cell through mitosis as it mediates the segregation of chromosomes, the orientation of the cleavage furrow, and the progression of cell division. Birth defects and tissue-specific cancers often result from abnormalities in mitotic events. Here, we report a proteomic study of the mitotic spindle from Chinese Hamster Ovary (CHO) cells. Four different isolations of metaphase spindles were subjected to Multi-dimensional Protein Identification Technology (MudPIT) analysis and tandem mass spectrometry. We identified 1155 proteins and used Gene Ontology (GO) analysis to categorize proteins into cellular component groups. We then compared our data to the previously published CHO midbody proteome and identified proteins that are unique to the CHO spindle. Our data represent the first mitotic spindle proteome in CHO cells, which augments the list of mitotic spindle components from mammalian cells.

## Introduction

Segregation of genetic material is indispensible for the propagation of all species. Each cell relies on a dynamic microtubule-based machine called the mitotic spindle to facilitate the cell division process [1], [2], [3]. Failures in mitosis can lead to birth defects, various leukemias, and tissue-specific tumors [4], [5], [6], [7], [8], suggesting that knowledge of the molecular make-up of the mitotic spindle is central to our understanding of a variety of human diseases. Many factors that regulate mitotic spindle function and cell division have been identified using genetic and biochemical methods over the past forty years [9], [10], [11], [12], [13]. Recently, genomic and proteomic screens have added to the growing number of mitotic and cell division factors, yet many of the components necessary for mitotic spindle function and cell division still remain unknown [9], [14], [15], [16], [17], [18], [19], [20], [21], [22], [23]. In order to understand how this macromolecular machine drives mitosis, the field has started to catalog all components in mitotic structures and construct networks of protein interactions. In this way, genomic and proteomic approaches will continue to enhance our overall understanding of mitotic spindle function and directly contribute to our knowledge of numerous human disease pathologies.

The mitotic spindle is a complex, microtubule-based structure that facilitates the separation of chromosomes and plays an essential role in cytokinesis [1], [9], [24], [25]. Spindle microtubules attach to specific sites on the chromosomes called kinetochores and are anchored by complex structures called centrosomes at each end, forming a bipolar spindle [24], [26], [27]. Multiple microtubules connect the centrosome to the kinetochore, creating a stable link to the chromosomes at the metaphase plate [24], [25]. Signals from the mitotic spindle also dictate where the acto-myosin ring and cleavage furrow will form at the cell cortex [1], [9], [12], [28], [29], [30], [31]. As mitosis progresses into anaphase and telophase, part of the spindle transforms into the central spindle, which is comprised of overlapping, anti-parallel microtubules [1]. The central spindle is then bundled by the ingressing furrow into the midbody [1], [9], [12], [30], [31]. Successful cell division depends on the coordination of microtubules, actin, and membrane to produce two daughter cells, each with its own complement of the genome.

Numerous proteins regulate the strength and dynamic nature of the mitotic spindle structure. NuMA and TPX2, for example, bind to microtubules and focus the spindle pole by maintaining the tie between microtubules and centrosomes [25], [32]. PRC1 bundles microtubules in the spindle midzone, which reinforces the strong link to the chromosomes [32]. Motor proteins, such as dynein and multiple kinesins, direct spindle orientation and generate force to direct movement of the tethered chromosomes to opposite poles of the cell [33], [34], [35], [36]. Regulation of mitotic progression is provided by several kinases, such as polo-like kinase 1 (PLK1), cyclin-dependent kinase 1, and aurora kinases A and B, which play roles in the assembly and movement of the mitotic spindle [18], [19], [21]. Studies of individual spindle components have revealed important pieces of information about how mitosis functions, yet a better understanding of their context in mitosis is necessary to fully understand their roles. Broader genomic and proteomic studies have begun to deepen our perspective on mitotic events.

In the past 12 years, proteomic analysis of spindles and spindle poles have identified numerous factors necessary for distinct steps in mitosis and spindle assembly and dynamics. One of the very first proteomic studies of spindle-associated proteins was of isolated *Saccharomyces cerevisiae* spindle poles. This pioneering work identified ten novel spindle pole components using 1–D gel analysis prior to mass spectrometry [14]. Subsequently, the *Drosophila* centrosome proteome project identified eleven additional proteins critical for centrosome stability [20]. Astrin, a protein which crosslinks microtubules in the spindle, was identified from mitotic HeLa cell extracts after 1–D gel excision of bands [15], [32]. Using mass spectrometry and sequence similarity database searching, the microtubule-associated proteome obtained from *Xenopus* meiotic egg extracts identified several components of the protein translation machinery, suggesting that protein translation may occur on the spindle [16]. In 2005, the first attempt to identify the entire mammalian mitotic spindle proteome was described for HeLa cells [17]. Drawbacks of all of these studies [14], [15], [16], [17], [23], however, are the gel extraction techniques prior to mass spectrometry analysis, known to lead to a significant loss of identified proteins [37], [38]. One or two-dimensional gel electrophoresis is commonly used to separate proteins before proteolysis and mass spectrometry analysis, but the technique has limited ability to detect low abundance proteins, membrane-associated proteins, and can lead to protein loss [39]. To avoid protein loss, our lab has implemented a MudPIT-based peptide separation via tandem liquid chromatography with direct mass spectrometry analysis [38]. Here, protein samples were directly subjected to MudPIT, bypassing the use of gel extraction techniques. MudPIT can resolve and detect proteins with a wide range of abundance and is efficient at detecting membrane-associated proteins [39], [40]. Work from our lab, in 2004, utilized this successful approach for the mammalian midbody proteome and identified numerous proteins involved in cytokinesis [9].

Here, we report the initial identification of the Chinese Hamster Ovary (CHO) cell mitotic spindle proteome, utilizing MudPIT, tandem mass spectrometry, and bioinformatic analyses. In total, we identified 1155 proteins in three out of four isolated spindle preparations. We categorized the sub-cellular location of each protein using Gene Ontology analysis and revealed 239 potential cell division factors associated with membrane, microtubule-associated, and actin-associated groups. Additionally, we compared the CHO spindle proteome to the CHO midbody proteome to identify 841 candidates specific for early events in cell division. In summary, our data represent the first mitotic spindle proteome in CHO cells, which augments our knowledge of the number of mammalian mitotic spindle components known to date. This knowledge ultimately plays a critical role in our understanding of human disease pathologies.

## Results

### Mitotic spindle isolation

**Figure 1:**
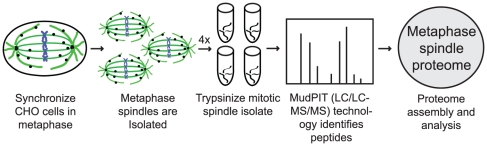
Proteomic strategy. CHO cells were synchronized at metaphase by successive thymidine and nocodazole blocks. Taxol was applied to stabilize the spindle structure. Cells were lysed in a hypotonic solution to release spindles. Four spindle samples were each trypsinized and MudPIT was performed. Proteins were identified by tandem liquid chromatography and tandem mass spectrometry (LC/LC-MS/MS), and the metaphase spindle proteome was assembled. Proteins in at least three mass spec runs appeared in the final list.

Our strategy to isolate and identify mitotic spindle proteins was as follows (Figure 1): First, we isolated mitotic spindles in four separate preparations from synchronized CHO cell populations. Second, metaphase-enriched samples were verified using immunofluorescence to assay for metaphase spindle structures. Third, the four metaphase-enriched samples were applied to two steps of liquid chromatography (LC/LC) and tandem mass spectrometry (MS/MS) analysis (LC/LC-MS/MS). Multi-dimensional protein identification technology, MudPIT, facilitated the identification of protein candidates in the complex protein mixture [9], [38].

**Figure 2:**
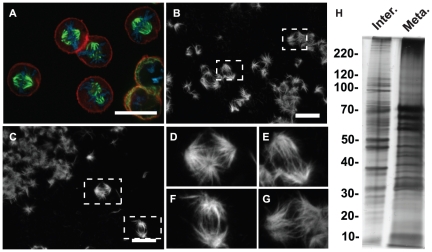
Synchronized CHO cells and isolated mitotic spindles. (A) CHO cells synchronized at metaphase by successive treatments of thymidine and nocodazole and stained for α–tubulin (green) and actin (red) and DNA (blue). (B–C) Isolated CHO spindles stained for α–tubulin. (D–G) Magnified view of isolated spindles from B–C (dotted boxes depict spindles shown in D–G). (D) A tetra-polar spindle. (E–G) Bipolar spindles. Scale bar indicates 20 µm. (H) Silver stained 1D gel depicting protein profiles from Interphase (Inter.) and Metaphase (Meta.) preps prior to MudPIT analysis.

The spindle isolation procedure was an adaptation of the midbody isolation preparation that has been published previously [9], [41], [42], and identical to the one used in Skop et al. [9], except for the time allowed for cells to progress through mitosis [9]. We synchronized CHO cells in metaphase by successive treatments of thymidine and nocodazole and assayed the cell population using immunofluorescence with anti-actin and anti-tubulin antibodies and DAPI [9], [41], [42] (Figure 2A). After releasing cells into fresh media, taxol and phalloidin were added to the cells to stabilize microtubule and actin structures, respectively [9], [42]. Phalloidin was included to maintain the similarity between the midbody and spindle isolation protocols. After the addition of taxol and phalloidin, cells were lysed, and the mitotic spindles and actin-associated structures were pelleted. To ensure that the correct structures were isolated, samples from each experiment were spotted on coverslips, fixed, and then stained with anti-tubulin antibodies to observe the spindle structure (Figure 2B–C). We observed both bipolar (Figure 2E–G) and splayed spindles in addition to tetra-polar spindles (Figure 2D), common for cells grown in tissue culture [43]. The mitotic spindles often clumped together in large groups, likely due to the ability of microtubules to adhere with each other in isolation [44], [45]. As the protocol used is imperfect, we also observed clumps of polymerized microtubules [9]. Interphase and spindle isolations were analyzed using silver staining (Figure 2H) to assay the consistency of each experiment, and reproducible banding patterns in each of our four sample preparations were observed [42]. Once the samples were verified using immunofluorescence and silver staining, each of four isolates was analyzed via tandem mass spectroscopy.

### MudPIT analysis

Each of our four metaphase spindle isolates was digested with trypsin, separated using tandem liquid chromatography (LC/LC), and analyzed using tandem mass spectrometry (MS/MS) and MudPIT analysis [38]. Peptides were eluted off columns, subjected to collision-induced dissociation, and spectra from fragment ions were recorded by mass spectrometry [38]. Since our protein samples were from Chinese Hamster Ovary cells, proteins were identified by comparing spectral data to known spectra found in a compiled mammalian database containing mouse, rat, and human FASTA sequences, similar to our previous approach [9]. Proteins were identified by prioritizing mouse proteins first, rat proteins second, and then human proteins last.

The raw data were sorted to assemble the final metaphase spindle list. A total of 1155 proteins were identified in at least three out of four samples at one or more peptide hit (Table S1). By applying this cutoff, we effectively reduced the estimated number of false positive hits in the list to 0. The list contains many known mitotic spindle components, such as tubulin subunits (TUBA1C, TUBB2B), the kinetochore protein CENPE, the cleavage furrow initiation protein RACGAP1, the cell cycle regulator PLK1, and the spindle pole protein NUMA1, which served as positive controls (Table S1). Over 85 known cell division factors were identified, 7% of the total proteins (Table S2). Some known spindle components, like aurora kinase B, are absent from our final list, but this is likely due to their low abundance, which has been reported previously [46]. In general, proteins in low abundance, large proteins, and charged peptides with modifications are difficult to detect by some mass spectrometry methods [47].

### Gene Ontology Analysis

**Figure 3:**
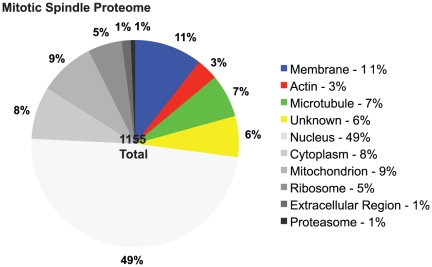
Subcellular location of metaphase spindle proteins. All proteins were categorized by the Cellular Component GO term using Gene Ontology analysis. Highlighted categories included proteins in GO categories associated with membrane, actin cytoskeleton, microtubule cytoskeleton, and uncharacterized proteins. Unknown indicates proteins that were not associated with a GO term and were not characterized by cellular component or localization in the literature as of January 2011.

To pinpoint membrane-cytoskeletal proteins from our large list of identified proteins, we determined the subcellular localization for each protein by applying Gene Ontology (GO) terms to our identified proteins (Figure 3, Table S2). For proteins associated with multiple GO terms (n = 664), we prioritized particular organelle localization over a general localization in the cytoplasm. The following cellular compartments were identified: nucleus, ribosome, mitochondrion, cytoplasm, extracellular region, membrane (plasma membrane, endosomes, Golgi, ER, endomembrane, membrane fraction), actin, or microtubule cytoskeleton (microtubules, microtubule cytoskeleton, spindle, kinetochore, centrosomes, microtubule organizing center). Due to the imperfect nature of the isolation protocol, some proteins, such as the mitochondrial and ribosomal proteins, may be associating with the mitotic spindle nonspecifically. However, further research would be necessary to eliminate them from the mitotic spindle proteome. Of our total of 1155 proteins identified, we determined that 11% were membrane-associated, 3% were actin-associated, 7% were microtubule-associated, 6% were unknown, 49% were nuclear, 9% were mitochondrial, 9% were cytoplasmic, 8% were ribosomal, 1% were proteosomal, and 1% were extracellular proteins (Figure 3). Of particular interest were the unknowns (74), membrane (122), actin- (39), and microtubule (78) -associated proteins, which made up 27% of the total proteins identified (313 proteins) (Figure 3). These 313 proteins were subsequently manually annotated and sorted using PubMed and UniProt. Membrane (11% of the proteins) was the largest group and included proteins tagged with GO terms such as membrane or vesicle and proteins linked to endo- and exocytosis (Figure 3). Proteins linked to actin and actin dynamics accounted for 3% of the proteins. For proteins not linking with Cellular Component GO terms to date and lacking localization data in the literature, we designated a group as unknown (Figure 3).

### CHO spindle proteome vs. CHO midbody proteome

**Figure 4:**
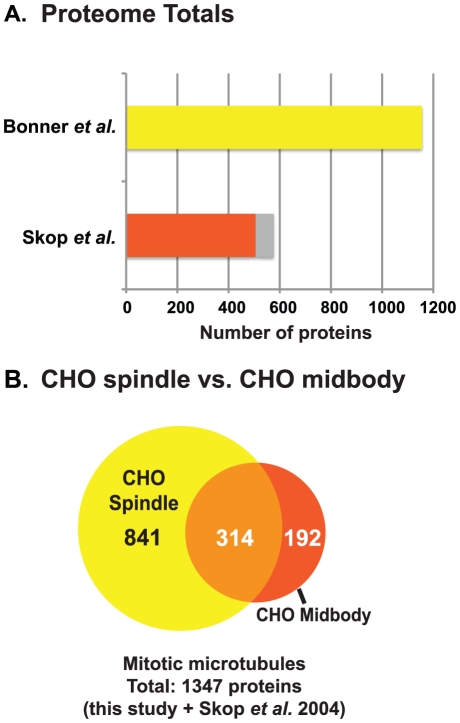
Comparison of metaphase spindle proteomes. (A) CHO mitotic spindle proteome is compared to the CHO midbody proteome [9]. Accession numbers were updated for Skop *et al.* [9], and an updated proteome total is indicated by red bar (n = 506). The gray bar represents the previous proteome total (n = 577). (B) Proteins unique to the CHO mitotic spindle proteome (n = 841) are shown in the yellow circle, proteins unique to the CHO midbody proteome (n = 192) are shown in the red circle, and the overlap represents the proteins in common (n = 314) [9]. The list of common and unique proteins is found in Table S3.

To identify proteins that may be specific to mitotic spindles, we compared the CHO metaphase spindle proteome (this study) to our published CHO midbody proteome (Figure 4) [9]. The spindle proteome contained 841 unique proteins, a group that may contain some proteins that function specifically during metaphase-anaphase transition or early in cytokinesis. We found that 314 proteins were in common between the proteomes, suggesting a possible function for these proteins throughout mitosis or cytokinesis (Figure 4 and Table S3) [9]. Proteins unique to the CHO mitotic spindle proteome included known spindle components such as SEPT7 and SEPT9, which are required for stable kinetochore localization of CENP-E [48], PAF1, which regulates Histone 2B and mediates the progression of the cell cycle [49], and MAGOHB, the mago-nashi homolog, which regulates cyclin dependent kinases [50]. Proteins appearing in only the CHO midbody proteome included ECT2 and KIF4A, both of which are critical for cytokinesis completion [51], [52]. Both lists contain substantial numbers of proteins in the mitochondrial and ribosomal groups, however, which could be associating indiscriminately with the microtubule structures. Combing both CHO cell data sets (spindle (this study) and midbody [9]), we have identified proteins that are present on mitotic microtubule structures in CHO cells.

### CHO spindle proteome vs. HeLa spindle proteome

In order to compile a core set of proteins that may comprise the mitotic spindle, we compared our data to the Sauer et al. HeLa spindle proteome [17]. Sauer et al. [17] identified 795 proteins associated with the HeLa mitotic spindle [17]. Interestingly, our study determined that 375 proteins were in common with the HeLa spindle proteome, and 780 proteins were unique to our CHO spindle proteome (Table S3) [17]. The spindle proteomes shared many cell division factors, such as CENPE, NUMA1, septin 2, several motor proteins, and multiple nucleoporins, for example. Proteins unique to our CHO mitotic spindle proteome included several membrane proteins, such as RAB7A, RAB14, and RAB31, which is not surprising given the success of membrane protein identification using MudPIT methods and the role of membrane trafficking in cell division events [2]. In addition, we identified 49% nuclear proteins, whereas the HeLa mitotic spindle proteome identified 21% nucleic acid binding proteins [17], likely due to their use of DNAse in their isolation assays, which we did not use. The protocols used to isolate the mitotic structures differed significantly in terms of DNAse treatment, latrunculin treatment, and intermediate filament removal, which may account for variation in proteins identified [17] (A.S., unpublished results). Advances in organelle isolation methods, mass spectrometry, and data analysis will continue to refine this growing list of mitotic spindle components.

## Discussion

Our data represent the largest number of spindle-associated proteins identified to date. We identified 1155 CHO cell spindle proteins using MudPIT analysis. Since microtubule-, actin-, and membrane-associated proteins play critical roles in mitosis, we highlighted 239 proteins that comprise these Gene Ontology categories as potential cell division factors. Skop et al. [9] demonstrated that a high percentage of the membrane trafficking and remodeling proteins found in the midbody play essential roles in cytokinesis completion [9]. Thus, we expect that some membrane proteins associated with the mitotic spindle are also critical for cell division. We compared our data to the CHO midbody proteome and found 314 proteins in common and 841 proteins unique to the CHO spindle [9]. The spindle-only subset could contain factors that function early in cell division. Our data not only improves data on the mitotic spindle proteome but also enhances our overall understanding of mitosis.

Among the 80 known cell division factors that our study identified were several nucleoporins. Interestingly, we observed an increase in the number of nucleoporins associated with the mitotic spindle compared to previous studies [53], [54], [55]. Nucleoporins act as the gatekeepers of the nucleus, and during mitosis, a number of nucleoporins associate with the mitotic spindle [55]. Several of the nucleoporins play roles in chromosome segregation, kinetochore-microtubule attachment, mitotic spindle morphology, and the regulation of microtubule polymerization at the kinetochores [53], [54], [56]. We identified 24 nucleoporins in the CHO mitotic spindle proteome, and 9 are not yet implicated in mitosis or cytokinesis. These nucleoporins may play novel roles in mitosis progression and represent a growing category of multifunctional proteins.

Membrane proteins represent a large group of potential mitotic factors. Some of these 122 proteins are known mitotic spindle components, such as clathrin heavy chain [17], [57]. However, most of these proteins lack described roles in mitosis. The four Bin/Amphiphysin/Rvs (BAR) proteins identified, TRIP10, ARHGAP17, sorting nexin 2, and sorting nexin 6, have not been associated with the mitotic spindle previously. BAR domain proteins play a key role in actin dynamics and endocytosis [58], [59], and their presence in the mitotic spindle preparation supports the connection between the mitotic spindle and membrane dynamics during cell division. Cell division requires the coordination of the cytoskeleton and the plasma membrane to separate chromosomes at the right time and place. It is not surprising that the mitotic spindle contains a variety of such proteins.

Kinases perform multiple functions in the cell as regulators of physiology and cell division [8], [60], [61]. We identified 38 kinases in the spindle proteome, several of which are well known mitotic regulators, such as polo kinase I (PLK1) and aurora kinase A [8], [62], [63], [64]. Many of the other identified kinases, such as hexokinase II and mitogen-activated protein kinase kinase kinase kinase 4, are associated with metabolic processes not necessarily required for cell cycle progression and cytokinesis [65], [66]. However, some kinases are multi-functional, straddling roles in routine cellular physiology and cancer prevention [60], [61], [67]. Phosphoglycerate kinase 1, which has not been associated with the mitotic spindle previously, is essential for glycolysis and also prevents angiogenesis in tumors [61]. Nucleoside diphosphate kinase A catalyzes phosphorylation of nucleosides and suppresses metastasis of primary tumors [67]. Several of the kinases found here are largely unexplored in the context of mitosis. Phospho-proteomic studies of the mitotic spindle in HeLa cells report many potential sites that these kinases could be regulating in the progression of cell division [18], [19], [21]. The 38 kinases identified in this study represent a pool of potential targets for cancer therapeutics and may indeed regulate cell cycle progression.

Comparative proteomics offers a way to monitor protein profile differences during the cell cycle. The CHO (this study) and HeLa (Sauer et al. [17]) spindle proteomes have 375 proteins in common [17]. We expected differences between the two proteomes, given that replicate analyses of samples from the same protocol often have only 60% overlap [47]. Additionally, since CHO and HeLa cells are from two different species, the protocols used to isolate spindles from each cell type differed [17]. One difference between the protocols was the incorporation of DNAse treatment into the HeLa spindle isolation [17]. Preliminary isolation procedures tested on the midbody proteome using DNAse led to a significant loss of known proteins as assayed by mass spectrometer analysis (A.S.; unpublished work). In addition, Gene Ontology categorizations, annotation and addition of sequences to protein databases, and mass spectrometer instrumentation likely contributed to differences we observed. The HeLa spindle proteome was analyzed by a CAPLC nano-HPLC system coupled to a Q-TOF mass spectrometer, while our CHO spindle samples were subjected to LTQ 2-dimensional ion trap mass spectrometer analysis (our study and [17]). Combining data from the CHO (this study) and HeLa (Sauer et al. [17]) spindle proteomes will expand our knowledge of the mammalian mitotic spindle proteome. Our comparison offers an initial core set of mitotic spindle components that will be a starting list for future studies on this complex and dynamic structure.

Comparison of our CHO cell spindle proteome with the CHO cell midbody proteome revealed an extensive list of proteins in common and distinct to each [9]. The combined proteomes of the mitotic spindle (this study) and midbody contains 1347 proteins with 314 proteins appearing in both [9]. Since the spindle and the midbody are both microtubule-based structures involved in cell division, the number of proteins in common is not surprising. Also, sample preparation protocols for the mitotic spindle and midbody were identical, aside from timing to allow mitosis to progress. Proteins found in these two structures may play multiple roles in mitotic events. By uniting and comparing these proteomes, we have assembled an initial protein profile for mitosis in CHO cells.

Our initial protein profile of mitosis in CHO cells has implications for cancer research. Major spindle components have been identified as targets for cancer therapeutics, which has led to the development and success of Taxol, for example, an anticancer drug that inhibits microtubule dynamics [68], [69]. Much work has focused on well-known proteins such as the aurora kinases and polo kinases, resulting in clinical trials for some kinase inhibitors [70], [71], [72], but every additional mitotic kinase identified represents a new potential therapeutic avenue. The list of proteins associated with mitotic structures from multiple screens has led to an important catalog of proteins that can be investigated for roles in human diseases. As mass spectrometry and protein isolation procedures continue to improve, additional mitotic proteins will be identified. Mitotic proteins will continue to generate numerous avenues of research into the mechanisms that regulate mitosis.

## Materials and Methods

### Cell Culture

Chinese Hamster Ovary cells, (CHO-S Cells, #11619012 from Invitrogen), were maintained in Opti-MEM containing 5% FBS, 50 U/mL penicillin and 100 µg/ml streptomycin. Cells were grown at 37°C in 5% CO_2_ within a humidified incubator.

### Isolation of spindles

Metaphase spindle proteome purification was adapted from a midbody purification protocol developed by Skop et al. [9]. CHO cells were synchronized at 37°C by a thymidine (16 h) and a nocodazole (4 h) treatment, and metaphase spindles were isolated after five minutes in fresh phalloidin and taxol-containing media (5 ug/ml) to stabilize polymerized actin and tubulin. Phalloidin was included to maintain a consistent isolation protocol between the midbody and spindle proteomes [9]. For the same reason, we did not include DNAse in our spindle isolation protocol. Cells were lysed in a hypotonic buffer that included 0.25% Triton X-100, 2 mM PIPES pH 6.9, and 20 ug/ml taxol, and spindles were pelleted at 2000 x g in 40% glycerol. Proteins samples were purified and concentrated by a chloroform precipitation.

### Gel electrophoresis and silver staining

Samples were run on a homemade 10% SDS-PAGE gel, washed with methanol (50% and 5%), and stained with silver nitrate at room temperature. Sample Buffer contained 1% SDS, 11% glycerol, 11% BME, 0.11% Bromophenol Blue and 0.05 M Tris pH 6.8.

### Immunofluorescence microscopy

Synchronized mitotic cells or isolated spindles were spotted onto coverslips and fixed for 15 minutes at room temperature in a formaldehyde/glutaraldehyde solution (3.7% formaldehyde, 0.3% Triton X100, 0.1% glutaraldehyde, 1X PHEM, pH 7.0). Coverslips were rinsed with PBS three times for five minutes, quenched twice for five minutes with PBS and a pinch of sodium borohydride, and washed with PBST three times for five minutes. After blocking for an hour with PHEM-Block, coverslips were incubated with primary antibody and PHEM-Block for an hour at 37°C. Reagents included: mouse monoclonal anti-alpha tubulin, #691251 from MP Biomedicals (microtubules); mouse monoclonal anti-actin, #MABR1501R from Millipore; Vectashield with DAPI, #H-1200 from Vector labs; TOTO-3, #T3604 from Invitrogen. Coverslips were washed three times for five minutes with PBST, and then incubated for another hour with the secondary antibody and PHEM-Block at 37°C. Coverslips were washed with PBST, stained with TOTO-3 in PBST, and washed again with PBST. Coverslips were mounted onto slides with Vectashield with DAPI.

### Sample Preparation for Mass Spectrometry Analysis

Sample digestion began when 60 µL of a buffer solution (8 M Urea, 100 mM Tris, pH 8.5) was added to solubilize the proteins previously TCA precipitated. To reduce the mixture, 0.3 µL of 1M TCEP (5 mM TCEP final concentration) was added and incubated at room temperature. To alkylate the sample, iodoacetamide (1.2 µL) was added (10 mM final concentration). For 15 minutes the sample was incubated at room temperature in the dark. Endoproteinase Lys-C (0.1 µg/µL) was added in 1.0 µL and shaken for 4 hours while incubating in the dark at 37°C. To dilute the solution to 2 M Urea, 180 µL of 100 mM Tris pH 8.5 was added. Calcium chloride (100 mM) was then added (2.4 µL) to reach a final concentration of 1 mM CaCl_2_. Trypsin (0.5 µg/µL) was added in the amount of 4.0 uL. For 12 hours the resulting mixture was shaken and incubated in the dark at 37°C. Formic Acid (90%) was added (15 µL) to neutralize the solution (final concentration 5% Formic Acid). The samples were centrifuged for 30 minutes using a 2°C table top centrifuge [73].

### Multidimensional Protein Identification Technology (MudPIT)

Following digestion, the proteins were pressure-loaded onto a fused silica capillary desalting column containing 3 cm of 5-µm strong cation exchange (SCX) followed by 3 cm of 5-µm C18 (reverse phase or RP material) pressure packed into a un-deactivated 250-µm inner diameter (i.d.) capillary. To complete sample assembly, a 100-µm i.d capillary consisting of a 10-µm laser pulled tip packed with 10 cm 3-µm Aqua C18 material (Phenomenex, Ventura, CA) was attached to the filter union (desalting column–filter union–analytical column). The resulting split-column was placed inline with a Hewlett Packard Agilent 1100 Quaternary Pump (Version 1.4; Palo Alto, CA) and analyzed using a customized 4-step separation method (90, 120, 120, and 150 minutes respectively) [73].

Step 1 utilized only buffer A (95% water, 5% acetonitrile, and 0.1% formic acid) and buffer B (80% acetonitrile, 20% water, and 0.1% formic acid). It began with 5 min of 100% Buffer A, followed by the following buffer B gradients: 5 min of 0-10%, 40 min of 10–45%, and 10 min of 45–100%. Twenty minutes of 100% buffer B ensued and the gradient program ended with 10 min of 100% buffer A. Steps 2–4 utilized Buffers A, B, and C (500 mM ammonium acetate, 5% acetonitrile, and 0.1% formic acid). Steps 2 and 3 each began with: 3 min of 100% buffer A, a 1 min gradient of 0 – X% buffer C, 7 min of X% buffer C, a 1 min gradient of 0 – 100% A, a 3.2 min gradient from 0 – 10% buffer B, a 74.8 min gradient from 10–45% buffer B, and then a 5 min 45–100% buffer B gradient. Ten minutes of 100% buffer B and a 5 min 0–100% gradient of buffer A followed. The sequence ended with 10 min of 100% buffer A. The buffer C portions consisted of 20% for step 2 and 50% for step 3.

Step 4 began in a similar fashion: 3 min of 100% buffer A, a 1 min gradient of 0–100% buffer C, 7 min of 100% buffer C, a 1 min gradient of 0–100% buffer A and a 8 min gradient from 0 – 10% buffer B. Its 10–45% buffer B gradient lasted for 85 minutes and the 45–100% buffer B gradient was for 10 min. Ten minutes of 100% buffer B and then a 15 min gradient of 0–100% buffer A ensued with the run ending with 10 min of 100% buffer A.

As a result of increasing the salt concentration (buffer C), the peptides will subsequently “bump” off of the SCX and then with a gradient of increasing hydrophobicity (buffer B) elute from the RP into the ion source. A distal 2.5 kV spray voltage was applied to elute the peptides from the microcapillary column. This applied voltage caused the peptides to directly electrospray into an LTQ 2-dimensional ion trap mass spectrometer (ThermoFinnigan, Palo Alto, CA). For each step of the multidimensional cycle, one full-scan mass spectrum (400–2000 m/z) occurred followed by 5 data-dependent MS/MS spectra at 35% normalized collision energy. The aforementioned HPLC solvent gradients and MS functions were all controlled by the Xcalibur data system Version 1.4 [73].

### Analysis of Tandem Mass Spectra

As each step was executed, the spectra were recorded to a RAW file. This data was then converted into .ms2 format through the use of RawXtract (Version 1.9.9). The MS/MS spectra were searched with the ProLuCID algorithm [74] against a FASTA database that contains European Bioinformatics Institute IPI human, mouse, and rat databases ([ftp://ftp.ebi.ac.uk/pub/databases/IPI], released in March 2010). A decoy database in which the sequence for each entry in the original database was reversed was concatenated to the FASTA database to estimate false discovery rate [75]. The ProLuCID search employed half tryptic search in terms of enzyme specificity and the final data set was filtered using the DTASelect (version 2.0.37H) program [76], [77]. DTASelect 2.0 uses a linear discriminant analysis to dynamically set XCorr and DeltaCN thresholds for the entire data set to achieve a user-specified false positive rate (1% at the protein level in this analysis). Proteins identified by the same peptide sets were clustered together by DTASelect 2.0. The DTASelect 2.0 program assembles identified peptides into proteins and protein groups by using a parsimony principle in which the minimum set of proteins accounts for all the observed peptides. A protein was deemed acceptable as a confident match based on the minimum number of one peptide with confidence 0.999 or higher. This dataset was then further filtered to remove contaminants (i.e. keratin) through the use of the “-e” (excludes protein names matching) and “-l” (excludes protein descriptions matching) commands.

### Proteome Assembly

After entries are compiled into a database that contains human, mouse, and rat proteins and filtered with DTASelect2, the ProteinCompare program was used to compare proteins identified in the 4 datasets. ProteinCompare removed redundant protein entries by selecting one representative protein for each protein group. Proteins identified with the same set of peptides were reported as one protein group by DTASelect2. A representative protein was selected for each protein group based on the following prioritization: annotated by GOA, mouse (Tax_Id = 10090) protein, rat (Tax_Id = 10116) protein, human protein (Tax_Id = 9606), and protein length. The proteins identified in 3 or more samples were reported in Table S1. After applying this additional filter, the estimated false positive rate is 0, i.e., no decoy hit was identified in 3 or more experiments.

### Updating accession numbers

The accessions (symbols) from the DTAselect output were updated using the Mouse Genome Database (MGD) at the Mouse Genome Informatics website, The Jackson Laboratory, Bar Harbor, Maine, World Wide Web [http://www.informatics.jax.org] (12/08/2010) [78], the Rat Genome Database (RGD), Rat Genome Database Web Site, Medical College of Wisconsin, Milwaukee, Wisconsin, World Wide Web [http://rgd.mcw.edu/] (12/08/2010) or using RGD Mart, dataset 20101119, [http://biomart.mcw.edu:9999/biomart/martview/] (12/08/2010) [79], or the HGNC Database, HUGO Gene Nomenclature Committee (HGNC) [80], EMBL Outstation - Hinxton, European Bioinformatics Institute, Wellcome Trust Genome Campus, Hinxton, Cambridge, CB10 1SD, UK [http://www.genenames.org/] (12/08/2010). The corresponding approved names are indicated. Any uncertainties were resolved by going back to the IPI accession number and comparing the sequence, searched for first at the European Bioinformatics Institute [http://www.ebi.ac.uk/Tools/dbfetch/dbfetch], with BLASTP versus current mouse, rat, or human RefSeq proteins at NCBI [http://blast.ncbi.nlm.nih.gov/Blast.cgi]. Systematic elimination of pseudogenes was performed manually. All identified pseudogenes were checked versus the DTAselect output, and IPI accessions were replaced as the representative protein if other non-pseudogenes were identified as part of the protein group. Any protein hits corresponding to only pseudogenes were identified by MGD, RGD, or HGNC and are indicated by asterisks in Table S1. A few hits that potentially identified read-through products (TUT1;EEF1G, INS;INS-IGF2, NME1-NME2;NME1;NME2, MC1R;TUBB3, RP11-74E24.2;ZC3H11A) were also manually rechecked versus the DTAselect output, and these were replaced as the representative protein if other non-read-through matches were identified as part of the protein group.

Proteins were referenced to HGNC approved genes in order to remove redundancy resulting from multiple protein isoforms from one gene and also from orthologous genes from two or more taxa appearing in the original protein set. Human homolog information was taken from MGD and RGD. Protein hits missing homolog information in these databases were manually analyzed using Treefam [http://www.treefam.org/] [81]. If a homolog could be identified, current approved symbol and approved name information as of the preparation of this manuscript on 12/10/2010 is listed in Table S2, from HGNC database [http://www.genenames.org/cgi-bin/hgnc_search.pl]. The five pseudogenes marked by an asterisk in Table S1 were not assigned a GO term and were removed from Table S2.

### GO Term Analysis

All of the representative proteins were subjected to gene ontology analysis with GoAssigner, developed in the Yates lab. The GoAssigner program assigned cellular component, molecular function, and biological process GO terms that were of interest, listed in Results and Table S2, based on GOA gene association files for human, mouse, and rat (March 2010 release of European Bioinformatics Institute [www.ebi.ac.uk/GOA/]).

GO Term assignments were manually verified for all candidate proteins and a selection of other proteins in the master list (n = 529). Each of those proteins was researched in the literature via PubMed [http://www.ncbi.nlm.nih.gov/pubmed/] for localization based on the exact gene name and aliases. Proteins were manually assigned the following groups based on subcellular location: nucleus, cytoplasm (which included some proteins localized in cytoplasmic organelles, such as peroxisomal proteins, where staining or expression indicated cytoplasmic localization), ribosome, mitochondrion, extracellular region, membrane (which included plasma membrane, endosomes, Golgi, ER, endomembrane, membrane fraction), actin, or microtubule cytoskeleton (microtubules, microtubule cytoskeleton, spindle, kinetochore, centrosomes, microtubule organizing center). Localization was assigned if the majority of literature papers were in agreement. In the case of dual subcellular localizations, the stronger localization was chosen, with an added requirement of confirmation by another paper. The unknown class of proteins (n = 74) corresponded to proteins that had no localization data in the literature. If a protein was assigned a subcellular localization, it was also researched for a published description of a role in cell division, mitosis, cytokinesis, or cancer (indicated in Table S2).

### Comparative Proteomics

The lists of proteins used for the comparison contain more items than listed previously due to expansion out of gene clusters, for example, to allow the updating and comparison of current HGNC gene symbols. These lists of proteins were compared in Microsoft Excel 2011 using PivotTable.

The protein set for the CHO midbody was derived from the accession numbers in Table S1 and Table S2 from Skop et al. [9]. Original accession numbers were updated to more recent UniProt accessions (2/2010), and duplicates from different species or different protein isoforms were removed. The unique UniProt accessions were mapped to gene names using UniProt KB Unimart, UniProt dataset [82], and these gene names were confirmed manually as HGNC symbols using HGNC, with ambiguities checked using BLASTP of the sequence corresponding to the original accession number. Accessions that didn't map successfully in Unimart were manually analyzed using BLASTP against the human RefSeq protein set using sequences from the original accessions, combined with TreeFam.org data for the non-human UniProt accessions. HGNC symbols were updated again on 12/14/2010 before comparison with this paper's protein set.

The protein set for the HeLa spindle proteome is derived from the 1121 accession numbers in Sauer et al. supplementary table 1 column 2 [17]. Updating the 1116 UniProt accession numbers and 5 IPI accession numbers from 795 rows required several steps. Most proteins were updated to current UniProt accessions using UniProt retrieve. Duplicates were removed. Sequences for the IPI accession numbers and 16 defunct UniProt accession numbers were recovered from other sources on the web, and BLASTP against the human RefSeq protein set with a cutoff of at least 90% identity was used to update some of these accessions. The unique current UniProt accessions were mapped to HGNC symbols using UniProt ID mapping to HGNC IDs. Biomart, database Ensembl Genes 60, dataset GRCh37.p2 [http://uswest.ensembl.org/biomart/martview/] (12/13/2010) was used to convert the HGNC IDs to HGNC symbols. Accessions that didn't map successfully to HGNC using this approach were mapped to gene names using Uniprot KB Unimart, UniProt dataset (12/14/2010), and these gene names were confirmed manually using HGNC. The remaining accessions that didn't map successfully using either previous approach were manually analyzed using BLASTP against the human RefSeq protein set with sequences from the original accessions, combined with TreeFam data for the non-human UniProt accessions introduced during updating. Several of the UniProt accession numbers mapped to two or more HGNC accessions, for example because several proteins had identical sequence in the original accession.

## Supporting Information

Table S1 The CHO cell metaphase spindle proteome. IPI accession numbers are found in column A. Numbers in m13, m14, 15 m, and m19 columns represent peptide hits in individual mass spec runs. The status column represents in which mass spec runs each protein was found, and the occurrence column states whether a protein was found in 3 or 4 out of 4 mass spec runs. In column H, protein descriptive names and taxa are reported. Corresponding gene symbols are listed in the gene symbol column with semicolons separating indistinguishable proteins, and the MGD/RGD/HGNC approved names are reported in column J. Pseudogenes are denoted with an asterisk in column K, if the row contains only pseudogene(s). Redundancy is present in this table.
(XLSX)

Table S2 Non-redundant data for the metaphase spindle proteome and corresponding GO terms. In this table, species differences are not included as separate entries, isoforms are not included as separate entries, and pseudogenes are not included. In column A, semicolons separate indistinguishable proteins. HGNC approved names are reported in column B. Cellular Component GO terms are found in column C. GO terms were manually annotated and validated for proteins in membrane, microtubule, actin, and unknown categories. Details are described in the Methods. Manually annotated proteins published as involved with cell division or cancer are marked in column D.
(XLSX)

Table S3 Comparative proteomics data of cell cycle proteomes. The comparison was performed using current unique HGNC symbols derived from updated accession numbers (see Methods). Using our updated lists, we found proteins in common between the CHO mitotic spindle and midbody [9], which are reported in column A. Unique proteins to the CHO mitotic spindle, when compared to the CHO midbody [9], are found in column B. Proteins that are found in both the CHO mitotic spindle and HeLa mitotic spindle proteomes [17] are reported in column D. Unique proteins to the CHO mitotic spindle, when compared to the HeLa mitotic spindle [17], are found in column E.
(XLSX)
